# Imaging of Intracellular and Extracellular ROS Levels in Atherosclerotic Mouse Aortas *Ex Vivo*: Effects of Lipid Lowering by Diet or Atorvastatin

**DOI:** 10.1371/journal.pone.0130898

**Published:** 2015-06-22

**Authors:** Matias Ekstrand, Maria Gustafsson Trajkovska, Jeanna Perman-Sundelin, Per Fogelstrand, Martin Adiels, Martin Johansson, Lillemor Mattsson-Hultén, Jan Borén, Max Levin

**Affiliations:** 1 Department of Molecular and Clinical Medicine/Wallenberg Laboratory, University of Gothenburg and Sahlgrenska University Hospital, SE-413 45, Gothenburg, Sweden; 2 Department of Oncology, Sahlgrenska University Hospital, SE-413 45, Gothenburg, Sweden; 3 Department of Pathology, Malmö University Hospital, Lund University, SE-205 02, Malmoe, Sweden; IDIBAPS - Hospital Clinic de Barcelona, SPAIN

## Abstract

**Objective:**

The first objective was to investigate if intracellular and extracellular levels of reactive oxygen species (ROS) within the mouse aorta increase before or after diet-induced lesion formation. The second objective was to investigate if intracellular and extracellular ROS correlates to cell composition in atherosclerotic lesions. The third objective was to investigate if intracellular and extracellular ROS levels within established atherosclerotic lesions can be reduced by lipid lowering by diet or atorvastatin.

**Approach and Results:**

To address our objectives, we established a new imaging technique to visualize and quantify intracellular and extracellular ROS levels within intact mouse aortas *ex vivo*. Using this technique, we found that intracellular, but not extracellular, ROS levels increased prior to lesion formation in mouse aortas. Both intracellular and extracellular ROS levels were increased in advanced lesions. Intracellular ROS correlated with lesion content of macrophages. Extracellular ROS correlated with lesion content of smooth muscle cells. The high levels of ROS in advanced lesions were reduced by 5 days high dose atorvastatin treatment but not by lipid lowering by diet. Atorvastatin treatment did not affect lesion inflammation (aortic arch mRNA levels of CXCL 1, ICAM-1, MCP-1, TNF-α, VCAM, IL-6, and IL-1β) or cellular composition (smooth muscle cell, macrophage, and T-cell content).

**Conclusions:**

Aortic levels of intracellular ROS increase prior to lesion formation and may be important in initiation of atherosclerosis. Our results suggest that within lesions, macrophages produce mainly intracellular ROS whereas smooth muscle cells produce extracellular ROS. Short term atorvastatin treatment, but not lipid lowering by diet, decreases ROS levels within established advanced lesions; this may help explain the lesion stabilizing and anti-inflammatory effects of long term statin treatment.

## Introduction

Reactive oxygen species (ROS) are highly reactive molecules continuously produced by mitochondrial electron transport and by enzymes such as NADPH-oxidases, xanthine oxidases and lipoxygenases [1–3]. Physiological ROS levels perform important regulatory functions within cells and in tissues. Excessive ROS levels irreversibly damage proteins, lipids, carbohydrates, and DNA [3, 4]. High ROS levels are considered to promote atherosclerosis progression, and possibly initiation. However, measuring arterial ROS levels is difficult and fundamental questions regarding ROS in atherosclerosis remain to be elucidated [5, 6].

There is no non-invasive technique that allows arterial ROS to be determined *in vivo*. ROS has a half-life of milliseconds and thus analysis of fixed or frozen material may not reflect the *in vivo* situation. Also, available techniques to assess arterial ROS do not discriminate between intracellular and extracellular ROS [7]. Such discrimination is important because intracellular and extracellular ROS promote atherosclerosis development through different mechanisms. Excessive levels of intracellular ROS damage DNA, proteins, lipids and carbohydrates inside cells whereas extracellular ROS oxidize lipoproteins and activate collagen-degrading matrix metalloproteinases outside cells [1, 3].

We introduce a technique that allows real-time visualization and quantification of ROS in intact aortas incubated under *in vivo* like conditions. Our technique utilizes the ROS-sensitive luminescent probes luminol and isoluminol [8]. Luminol penetrates cell membranes but isoluminol does not. Sequential use of the probes makes it possible to analyze both intracellular and extracellular ROS levels. ROS-dependent luminescence emitted from the intact aorta is visualized using a photon counting camera.

In mice, different stages of atherosclerosis are typically present in different segments of the aorta [9]. Notably, the aortic arch (aortic root to left subclavian artery) is highly atherosclerosis prone whereas the descending thoracic aorta (left subclavian artery to the diaphragm) is much more atherosclerosis resistant. We analyzed the aortic segment from the root to the diaphragm to visualize ROS in atherosclerotic and non-atherosclerotic segments of the same aorta.

Both intracellular and extracellular ROS has been suggested to promote early atherosclerosis development [1, 3]. If this is correct, increase in ROS levels would precede lesion development. However, it is not known at which point in atherosclerosis development aortic ROS levels increase. To address this question, we analyzed intracellular and extracellular ROS levels within the descending thoracic aorta at different stages of early atherosclerosis development.

In humans as well as in animal models of atherosclerosis, reduction of plasma lipids promotes regression and stabilization of advanced atherosclerotic lesions [10]. Reduction of arterial ROS levels may promote some of the beneficial effects of lipid lowering [11, 12]. To investigate this, we studied how lipid lowering, by diet or with atorvastatin, affects ROS levels within advanced atherosclerotic lesions in the aortic arch.

## Materials and Methods

### Experiment outline

This study consists of two parts:

1. We established methodology to assess intracellular and extracellular ROS in intact mice aortas under *in vivo*-like incubation conditions. Then, we used this method to::
  - Assess intracellular and extracellular ROS in early atherosclerosis by analyzing the relatively atherosclerosis resistant descending aorta (left subclavian artery to diaphragm) in mice fed either chow diet or atherogeic diet (for 3 or 7 weeks).
  - Assess intracellular and extracellular ROS in advanced atherosclerotic lesions by analyzing the highly atherosclerosis prone aortic arch (aortic root to left subclavian artery). ROS levels within the aortic arch were then tested for correlation to smooth muscle cell and macrophage content. Mice were fed either chow diet or atherogenic diet for 3, 5, 7, or 9 weeks to induce a wide range of lesions in the aortic arch.
2. In the second part of the study, we investigated the effect of lipid lowering, by diet or atorvastatin, on ROS levels in advanced atherosclerotic lesions. To this end we assessed ROS in the atherosclerosis prone aortic arch of mice fed atherogenic diet for 7 weeks. 7 weeks of atherogenic diet was used to ensure development of advanced lesions in the aortic arch of all animals. Lipids were lowered by atorvastatin (5 final days) or by switching to chow diet (5 final days). The short duration of lipid lowering was chosen to avoid statin induced reduction of inflammation within atherosclerotic lesions [13, 14]. We wanted to avoid such an anti-inflammatory effect because it would obscure interpretation of a potential direct antioxidant effect of statin treatment.

### Animals

Female *Apoe* ^*-/-*^ mice (n = 67, 36 in part one and 31 in part two) were used and atherosclerosis was induced by Western atherogenic diet [21% anhydrous milk fat (butterfat), 34% sucrose, and a total of 0.2% cholesterol, Harlan Teklad Inc., MA, USA]. All animal procedures were approved by the animal ethics committee at the University of Gothenburg and conform to the guidelines from Directive 2010/63/EU of the European Parliament on the protection of animals used for scientific purposes. At the end of experiments, mice were sacrificed using isofluorane and removal of the heart.

### Lipid lowering

Lipid lowering was achieved by either diet or pharmacologic treatment with atorvastatin. Lipid lowering by diet was achieved by switching atherogenic Western diet to chow diet for 5 days. Atorvastatin (dissolved in DMSO and carboxymethyl cellulose) or vehicle (DMSO and carboxymethyl cellulose only) was given once daily by gavage for 5 consecutive days. To extrapolate human doses of atorvastatin into mouse doses, we used pharmacokinetic allometric scaling based on body surface area [15]. The maximum dose of atorvastatin in humans, 80 mg/day (0.5 to 1.6 mg/kg in the patient weight range of 50–150 kg), translates to a mouse dose of 7–20 mg/kg. Each animal received five doses of atorvastatin and steady state concentrations in plasma would be expected at the 3-4^th^ dose. To ensure high atorvastatin exposure with only five doses, we used a dose of 100 mg/kg.

### Tissue handling

Mice were anaesthetized using isoflurane. The thoracic cage was opened and a blood sample was drawn from the right ventricle for plasma lipid analysis. Then, a syringe was inserted through the left ventricle and the aorta was perfused with PBS. A segment from of the aorta ranging from the distal aortic root to the diaphragm was removed by dissection. The aortic segment was carefully cleaned of periadventitial fat in a dissection microscope (Leica M2S, Leica microsystems AB, Kista, Sweden). Then, the aortic segment was put in organ bath and used for ROS analysis or snap frozen in liquid nitrogen for mRNA preparation. The aortic root and arch was embedded in OCT and frozen in isopentane chilled with liquid nitrogen and used for histological characterization of lesions.

### Extracellular and intracellular ROS *ex vivo*

**Fig 1:**
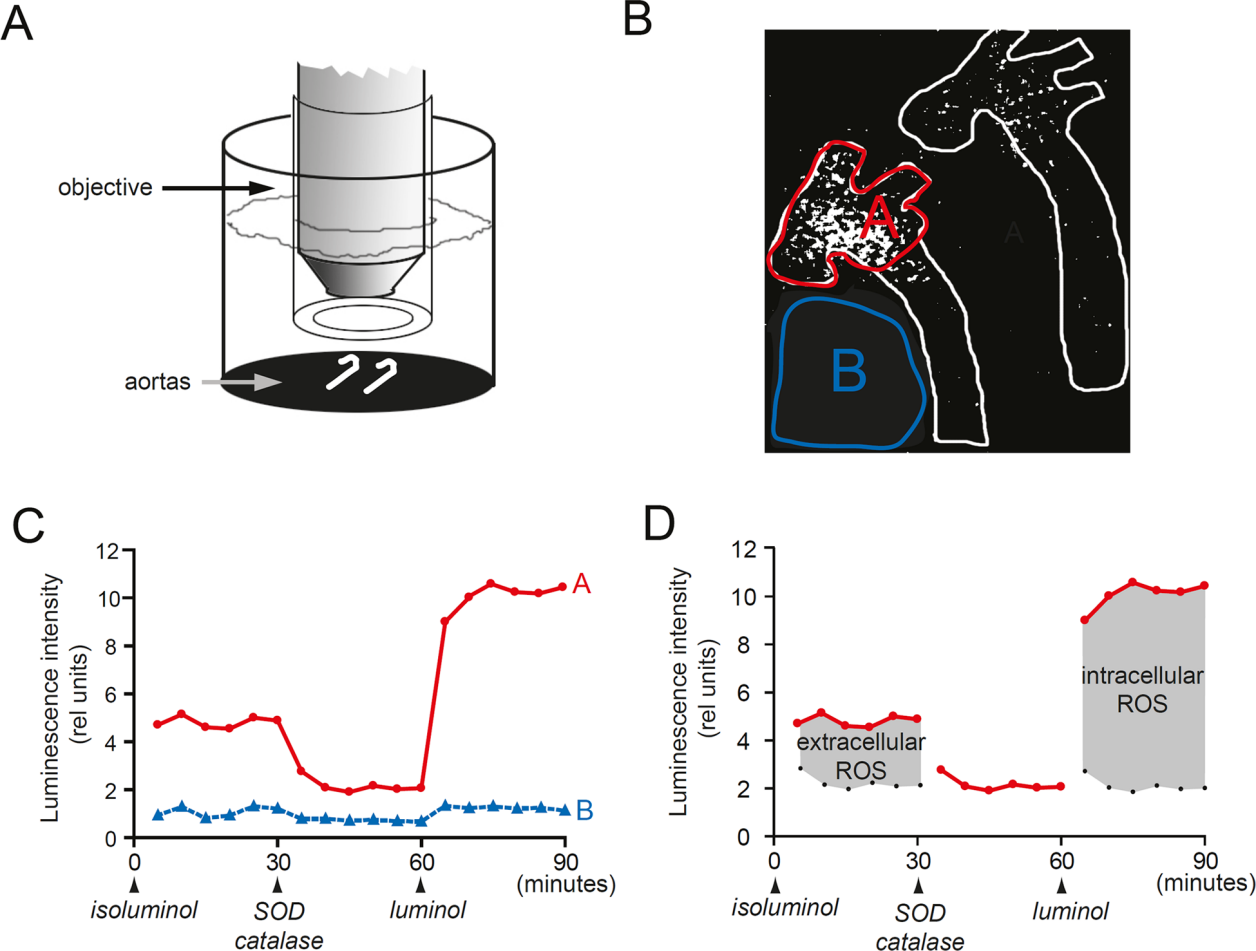
Real-time imaging of intracellular and extracellular ROS *ex vivo*. (A) Fresh aortas in organ bath were depicted through microscope optics connected to a photon counting camera. (B). Photon counting image of two aortas after addition of extracellular ROS probe isoluminol. The left aorta had extensive atherosclerosis and right aorta limited atherosclerosis. (C) Luminescence intensity (y-axis) over time in atherosclerotic aortic arch of the left aorta (A—red line) and background (B—blue line). Time points for addition of isoluminol, SOD and catalase, and luminol is indicated at the y-axis. (D) Extracellular ROS levels (left grey area) were quantified by subtracting the SOD/catalase signal from the isoluminol signal. Intracellular ROS (right grey area) were quantified by subtracting the SOD/catalase blocked signal from the luminol signal.

Two freshly dissected aortas were put in organ bath with oxygenated (21% oxygen 5% CO_2_ in nitrogen) Krebs Ringer solution (Sigma-Aldrich, MO, USA) at 37°C. The experiment set up and a typical registration is shown in Fig 1.

First, a brightfield image of the aortas was obtained through a microscope objective (2 x /0.1 NA Nikon Apochromat, BergmanLabora AB, Danderyd, Sweden) immersed in the organ bath. The brightfield image was used to outline aortic structures in the subsequent photon counting images. Then, the light was turned off and three consecutive 30 minute photon counting images were obtained in complete darkness.

1. **Registration**: the cell membrane impermeable chemiluminescence probe isoluminol (0.1 mM, SigmaAldrich) was added to the Krebs Ringer solution. Isoluminol reacts, in the presence of peroxidase (Horseradish peroxidase, 8 U/ml, Worthington Biochemical Corp., NJ, USA), with extracellular ROS and photons are emitted. The emitted photons were registered by an ultra-low light sensitive photon counting camera (Hamamatsu Photonics Norden AB, Kista, Sweden).
2. **Registration**: extracellular ROS was blocked using superoxide dismutase (SOD, 50 U/ml, Worthington Biochemical Corp) and hydrogen peroxide scavenger catalase (50 mM, Worthington Biochemical Corp). SOD and catalase do not penetrate cell membranes and thus selectively blocks extracellular ROS.
3. **Registration**: the cell-permeable ROS probe luminol (0.1 mM, Sigma Aldrich) was added to measure intracellular ROS. Because extracellular ROS was blocked, the luminol-dependent photon emission corresponded to the local levels of intracellular ROS.

As a control, ROS were analyzed in atherosclerotic aortas with (n = 3) or without (n = 3) heat inactivation (80°C for 10 minutes).

Photon-counting images were obtained using Wasabi software (Hamamatsu Photonics Norden AB). Further analysis of images was performed using KS400 software (Carl Zeiss AB, Stockholm, Sweden).

### Aortic ATP content

ATP concentrations were measured to assess viability in aortas after ROS analysis. ATP levels in sections of snap frozen aortas were analyzed using bioluminescence imaging as previously described [16].

### Extent of atherosclerosis

In the descending aorta (left subclavian artery to diaphragm), which only developed limited atherosclerosis even in mice fed Western diet, the presence or absence of early atherosclerotic lesions was noted during microscope dissection. Early atherosclerotic lesions were seen as small white dots through the transparent media in the aorta.

In mice treated with atorvastatin or vehicle (n = 12), we quantified the percent of the descending thoracic aorta (aortic root to diaphragm) covered by atherosclerotic lesions. To this end, thoracic aortas were pinned out by *en face* technique and fixed in 70% ethanol for 5 minutes, stained with 0.5% Sudan IV for 6 minutes, and differentiated for 3 minutes in 80% ethanol. Aortas were photographed and the extent of atherosclerosis quantified using image analysis (KS400, Carl Zeiss AB).

### Lesion composition

To correlate lesion cell composition with ROS levels, aortic arches (aortic root to left subclavian artery) (n = 25) were frozen after ROS analysis. Frozen sections, 8 μm-thick, were taken from 4 levels of the aortic arch; aortic valves, right common carotid artery, left carotid artery, and left subclavian artery. In mice treated with atorvastatin or vehicle (n = 12), sections from the aortic root was used to analyze cell content. As an estimate of total smooth muscle cell and macrophage content within the aortic arch, tissue section from these levels were double stained for macrophages and smooth muscle cells. Digital images of tissue sections were obtained with a cooled CCD color camera (Axiocam, Carl Zeiss) mounted on an Axiophot 2 microscope (Carl Zeiss). Identical microscope and camera settings were used for all sections. Smooth muscle cells positive areas and macrophage positive areas were quantified as previously described [17] using a KS400 image-analysis system (Carl Zeiss). Data from all levels were combined to get an estimation of smooth muscle cell and macrophage content within the entire aortic arch.

Double staining was used to simultaneously demonstrate macrophages (rat anti-mouse Mac2, IgG2a, clone CL8942AP, 50 μg/ml, Nordic Biosite AB, Täby, Sweden) and smooth muscle cells (SMC) (polyclonal rabbit anti-human smooth muscle cell actin, 1 μg/ml, ab5694, Abcam, Cambridge, UK). Sections were incubated with anti-Mac2 at 4°C overnight and then with alkaline phosphatase-conjugated anti-rat IgG (Pharmingen AB, Stockholm, Sweden; 1:50) for 30 min. Anti-actin was added for 60 min, followed by biotinylated anti-rabbit IgG (Pharmingen AB; 1:50) and streptavidin-conjugated *β*-galactosidase (1:100) for 30 min each. Alkaline phosphatase activity (macrophages) was visualized using The Vector Fast Red kit (Bionordika AB, Stockholm, Sweden). *β*-Galactosidase (SMCs) was visualized with x-gal and ferricyanide.

T cells were identified by incubation with anti-CD4/CD8 antibody. Bound antibody was visualized with a fluorescent secondary antibody (Alexa 488 conjugated, donkey anti-rabbit IgG, 1:400, Life Technologies Europe BV). Nuclei was stained with Hoechst 33342 (Life Technologies Europe BV; 2 μg/ml) and sections were mounted using Prolong Gold mounting medium (Life Technologies Europe BV). Sections incubated with irrelevant rat and rabbit IgGs (Santa Cruz Inc., CA, USA) served as negative controls.

The number of T cells was determined by manual counting in the microscope at 400× magnification. Counts were expressed as cells/mm^2^.

### Aortic arch inflammation

To assess the degree of inflammation, aortas (n = 6) were perfused with PBS, rapidly cleaned of adventitial fat and the aortic arch was snap frozen in liquid nitrogen. Subsequently, the aortic arch was homogenized and mRNA was prepared. RT-PCR was used to determine mRNA levels of the following inflammation markers: CXCL 1, ICAM-1, MCP-1, TNF-α, VCAM, IL-6, and IL-1β. To allow comparisons, mRNA levels were normalized to mRNA levels of 18S.

### Plasma lipids

To analyze plasma cholesterol and triglyceride levels, blood samples were drawn from the right ventricle immediately after the thoracic cage was opened. Total cholesterol and triglyceride levels were measured in plasma samples using the Konelab 20 autoanalyzer (ThermoFisher Scientific Oy, Vantaa, Finland).

### Statistics

Data are mean ± SEM. Data distribution was assessed with normality test. Significant differences were tested with one way ANOVA with Dunnett’s multiple comparison test, one sample t-test or unpaired t-test. P-values less than 0.05 were considered significant. Graph Pad prism software was used throughout (GraphPad Software, Inc, CA, USA).

## Results

### Imaging of intracellular and extracellular ROS in intact aortas *ex vivo*

A typical experiment is shown in Fig 1. Two freshly dissected aortas from female *Apoe* ^*-/-*^ mice were put in organ bath (Fig 1A). To quantify intracellular and extracellular ROS levels, we performed three consecutive bioluminescence registrations (Fig 1B–1D). First we analyzed extracellular ROS using the cell-impermeable ROS probe isoluminol. After 30 minutes we added SOD and catalase to inactivate extracellular ROS. We then added the cell-permeable ROS-probe luminol to quantify intracellular ROS. ROS were analyzed separately in the atherosclerosis prone aortic arch and in the relatively atherosclerosis-resistant thoracic aorta.

As a control, ROS were analyzed in atherosclerotic aortas (n = 3) with or without (n = 3) heat inactivation (80°C for 10 minutes). Heat inactivation reduced ROS levels, intracellular and extracellular, to background levels.

We used incubation conditions previously shown to maintain *in vivo* like levels of energy metabolites and oxygen within atherosclerotic arteries [18]. ATP levels after incubation were at the same level as in snap frozen aortas (0.52 ± 0.14 vs 0.45 ± 0.09; n = 4 in each group).

### Elevated intracellular ROS precedes development of atherosclerotic lesions

**Fig 2:**
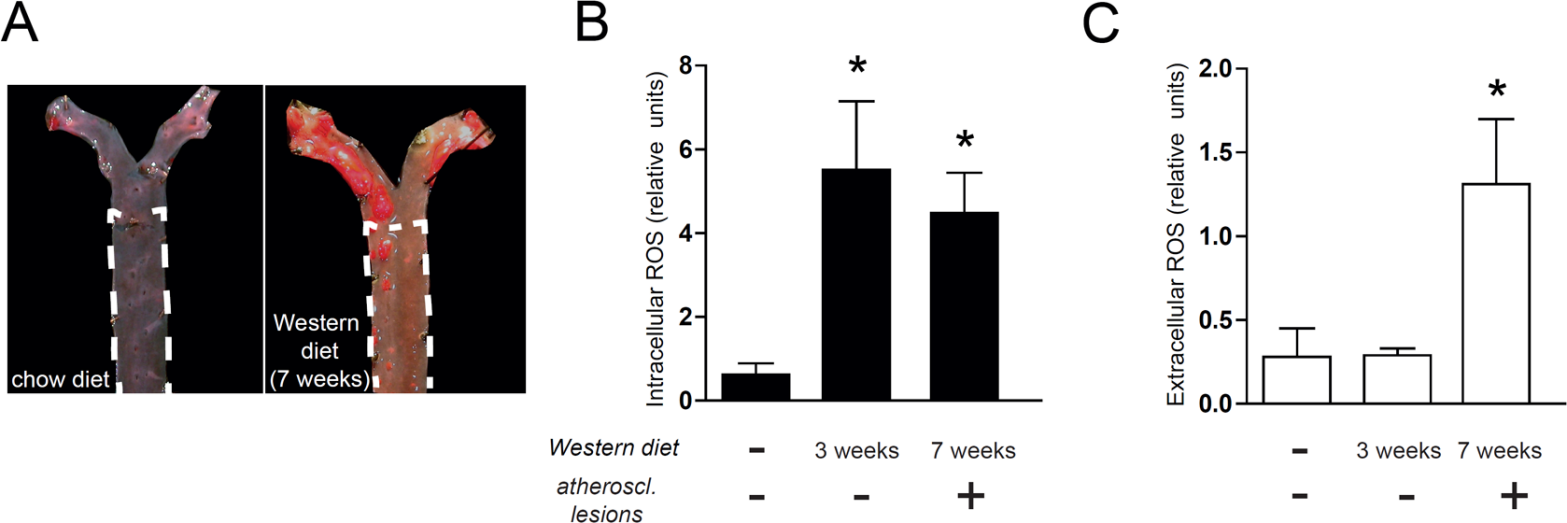
Intracellular ROS increase before formation of atherosclerotic lesions in hyperlipidemic mice Female *Apoe* ^-/-^ mice were fed either chow diet or Western diet. *(A)* The descending thoracic aorta (delineated by dashed line) was relatively spared from atherosclerosis, even in mice fed Western diet. *(B)* In the descending thoracic aorta, Western diet increased extracellular ROS before formation of atherosclerotic lesions *(C)* Extracellular ROS levels, in contrast, were increased after formation of atherosclerotic lesions. n = 6–7 in each group. *p>0.05 vs chow diet, One way ANOVA with Dunnett’s multiple comparison test.

To investigate if vascular ROS increase prior to or after lesion development, we fed *Apoe* ^*-/-*^ mice chow diet or Western diet for 3 or 7 weeks. No atherosclerotic lesions were detected in the descending thoracic aortas of mice fed chow diet or 3 weeks of Western diet. Scattered lesions were present in the descending thoracic aorta after 7 weeks of Western diet (Fig 2A). Intracellular ROS were increased already after 3 weeks of Western diet, before lesions were visible (Fig 2B). In contrast, extracellular ROS levels were increased after 7 weeks, when scattered lesions were present (Fig 2C). Thus, Western diet increases intracellular ROS before development of atherosclerotic lesions.

### Macrophages produce intracellular ROS and smooth muscle cells extracellular ROS in atherosclerotic lesions

**Fig 3:**
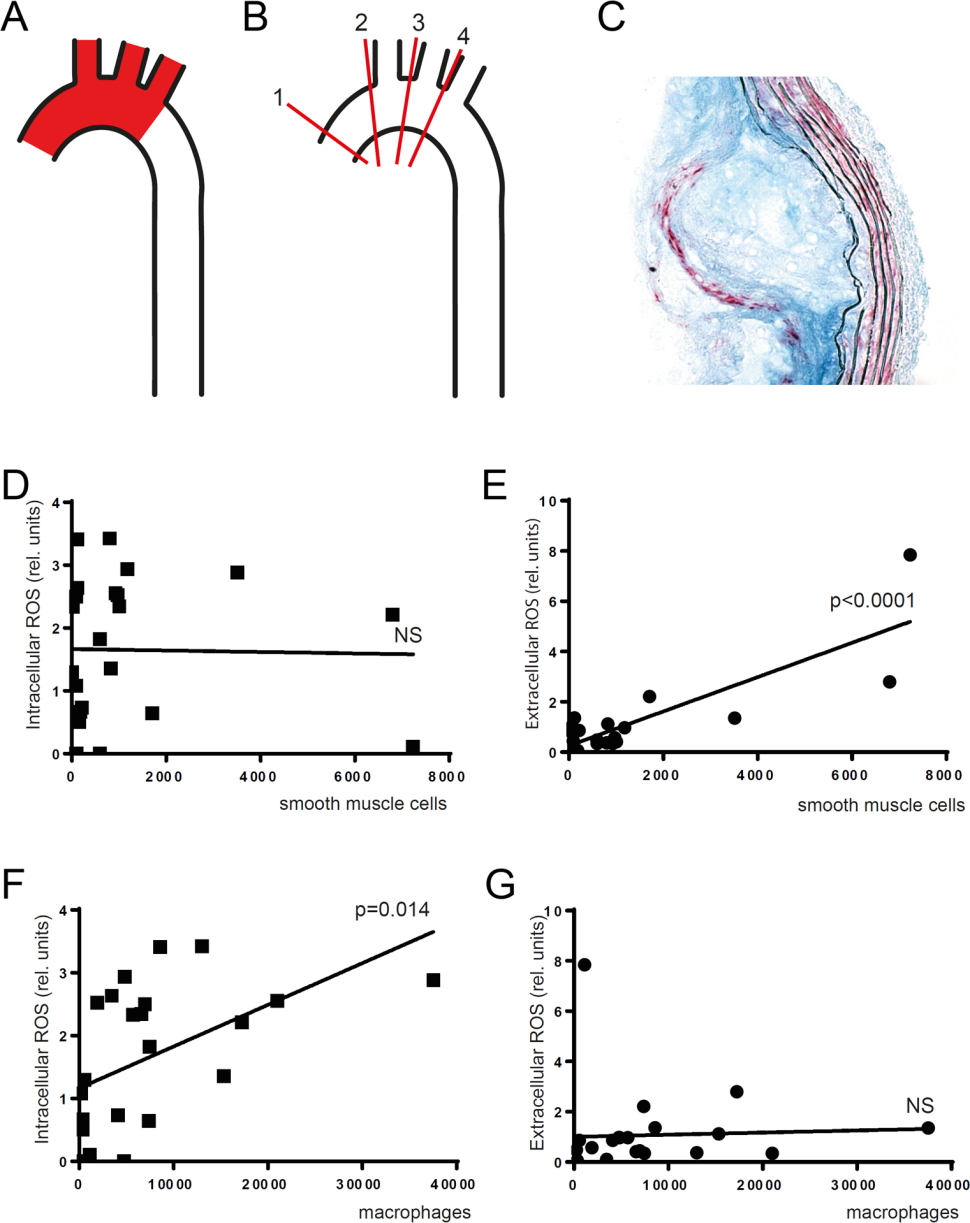
Intracellular ROS correlates with macrophage content and extracellular ROS with smooth muscle cell content in advanced atherosclerotic lesions Female *Apoe* ^*-/-*^ mice were fed Western diet to induce advanced atherosclerotic lesions in the aortic arch. (*A)* Intracellular and extracellular ROS were analyzed in the aortic arch (red area) *(B)* Smooth muscle cell and macrophage content was quantified in the aortic arch by analyzing sections from 4 different levels. *(C)* Section stained for macrophages (CD68: blue) and smooth muscle cells (α-actin: red). *(D and E)* Smooth muscle cell content in lesions correlated with extracellular *(E)* but not intracellular ROS *(D)*. *(F and G)* Macrophage content in lesions correlated with intracellular *(F)* but not extracellular ROS *(G)*. Linear regression (n = 25). NS (non significant).

Both smooth muscle cells and macrophages produce ROS in atherosclerotic lesions [1–3]. However, it is not known if the cell types produce intracellular and/or extracellular ROS. To investigate this, we correlated intracellular and extracellular ROS levels with cellular composition of atherosclerotic lesions in the aortic arch (Fig 3). *Ex vivo* levels of intracellular and extracellular ROS were assessed in the aortic arch of female *Apoe* ^*-/-*^ mice fed Western diet for 3–9 weeks. After ROS analysis, lesion content of macrophages and smooth muscle cells was quantified. Intracellular ROS levels in atherosclerotic lesions showed significant positive correlation with macrophage content. Extracellular ROS levels showed significant positive correlation with lesion smooth muscle cell content. This indicates that macrophages within atherosclerotic lesions predominantly produce intracellular ROS and smooth muscle cells extracellular ROS.

### High-dose atorvastatin reduces intracellular and extracellular ROS levels within atherosclerotic lesions

**Fig 4:**
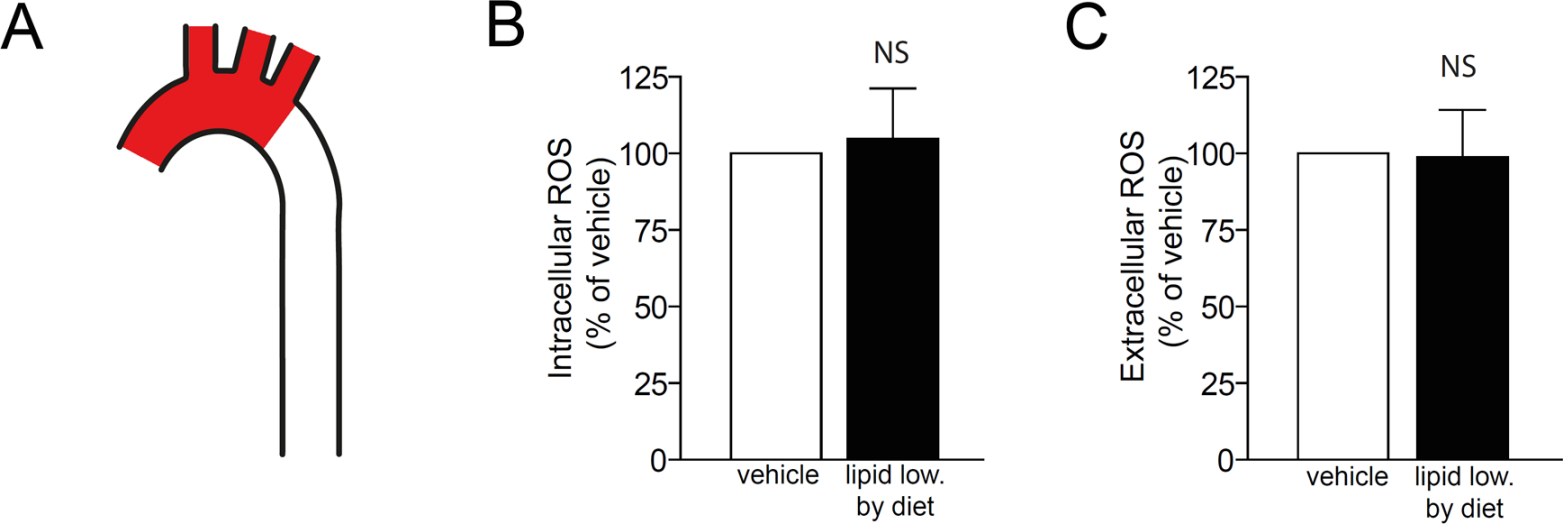
Lipid lowering by diet does not affect ROS levels within the atherosclerotic aortic arch Female *Apoe* ^*-/-*^ mice were first fed Western diet to induce advanced lesions in the aortic arch. Then, lipids were lowered by switching to chow diet for five days. A control group was maintained on Western diet. *(A)* Intracellular and extracellular ROS were assessed in the atherosclerotic arch (red). *(B)* Lipid lowering by diet did not affect intracellular ROS. *(C)* Lipid lowering by diet did not affect extracellular ROS. NS—non significant. One sample t-test. n = 6 in each group.

We then investigated if diet-induced or pharmacologically induced lipid lowering affects ROS in atherosclerotic lesions. We first tested if diet-induced lipid lowering reduces ROS levels in atherosclerotic lesions. To this end, one group of *Apoe* ^*-/-*^ mice was fed Western diet for 7 weeks and another group had Western diet replaced by chow diet for the last 5 days. Diet-induced lipid lowering did not change intracellular or extracellular ROS levels within lesions (Fig 4). Thus, short term lipid lowering does not influence ROS levels within atherosclerotic lesions.

**Fig 5:**
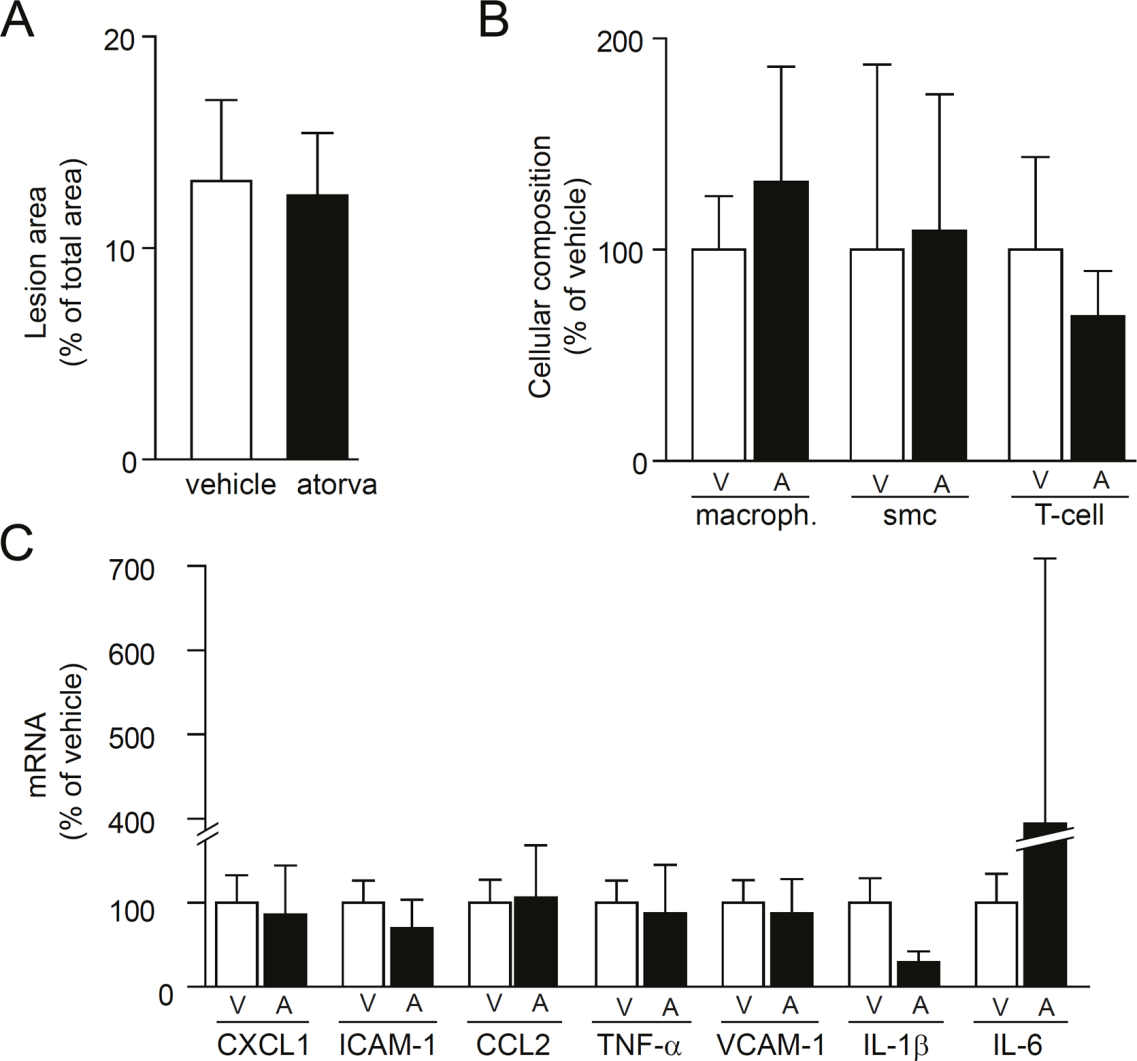
Atorvastatin does not affect extent of atherosclerosis, lesion cell composition or lesion inflammation Female *Apoe* ^*-/-*^ mice were first fed Western diet to induce advanced lesions in the aortic arch. Then, mice were treated with vehicle (DMSO) or oral atorvastatin (100 mg/kg per day) for 5 days. *(A)* Atorvastatin did not affect lesion area in the aorta. *(B)* Atorvastatin did not affect lesion cell composition. *(C)* Atorvastatin did not affect mRNA levels of inflammatory mediators. n = 6 in each group. Student’s t-test.

**Fig 6:**
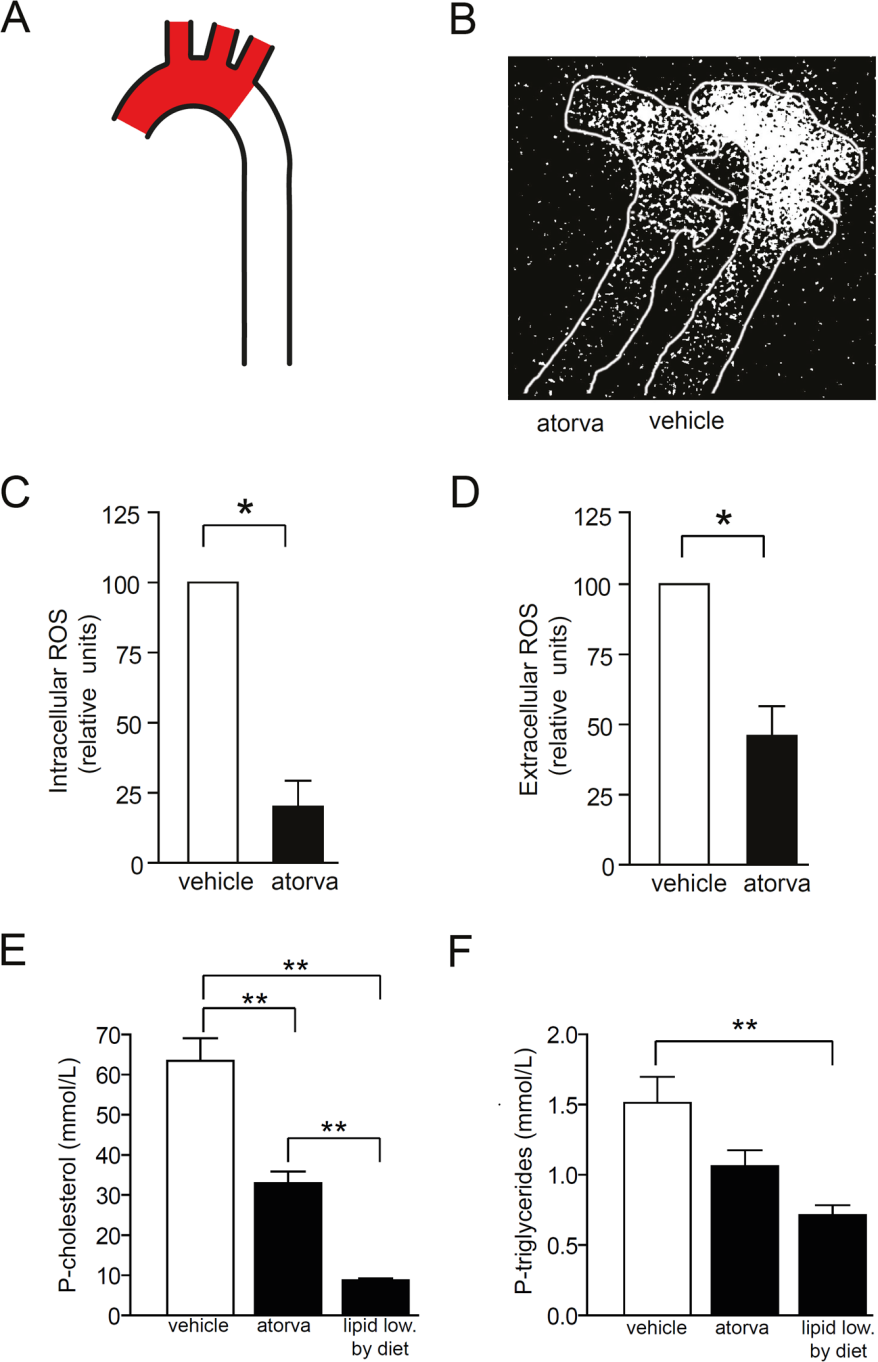
Atorvastatin reduces intracellular and extracellular ROS levels within the atherosclerotic aortic arch. Female *Apoe* ^*-/-*^ mice were fed Western diet to induce advanced atherosclerotic lesions in the aortic arch. Then, mice were treated with vehicle (DMSO) or oral atorvastatin (100 mg/kg per day) for 5 days. *(A)* ROS levels were analyzed in the aortic arch (red areas). *(B–D)* Atorvastatin treatment reduced intracellular *(B and C)* and extracellular ROS levels *(D)*. *(E and F)* Plasma cholesterol *(E)* and triglycerides *(F)* was reduced by atorvastatin and, to larger extent, by lipid lowering by diet. n = 6 in each group. **p<0.01 vs vehicle, *p<0.05 vs vehicle. One sample t-test (C and D). ANOVA with Dunnet’s test for multiple comparisons (E and F).

We then investigated if lipid lowering by atorvastatin intervention affects ROS levels within established atherosclerotic lesions. Atorvastatin was administered to *Apoe* ^*-/-*^ mice during the final 5 days on a 7 week Western diet. We used short treatment to avoid statin-induced changes in lesion composition since prolonged statin treatment is known to have anti-inflammatory and stabilizing effects on atherosclerotic lesions [13, 14, 19]. The short intervention with atorvastatin did not affect lesion area, lesion cell composition, or mRNA levels of inflammatory mediators in the aortic arch (Fig 5). However, atorvastatin significantly decreased both extracellular and intracellular ROS levels within the atherosclerotic aortic arch (Fig 6A and 6B). Interestingly, atorvastatin decreased plasma cholesterol and triglycerides less than diet induced lipid lowering (Fig 6C and 6D). Thus, atorvastatin treatment decreased ROS in atherosclerotic lesions but the effect was not linked to its lipid-lowering effect, changed lesion composition, or reduced inflammation.

## Discussion

In this study, intracellular and extracellular ROS levels were studied *ex vivo* in intact mice aortas. We show that intracellular ROS increase early in atherosclerosis development, prior to lesion formation. Extracellular ROS increased later, when lesions were established. Intracellular ROS correlated with macrophage content in lesions, and extracellular ROS with smooth muscle cell content. Furthermore, short term lipid-lowering with atorvastatin, but not with diet, decreased both intracellular and extracellular ROS levels within advanced atherosclerotic lesions. Thus, atorvastatin has an antioxidant effect within lesions that is not dependent on lipid lowering *per se*.

Our finding that Western diet increased intracellular, but not extracellular, ROS levels in the aortic wall prior to the appearance of atherosclerotic lesions is in line with the observation that mitochondrial DNA damage within aortic smooth muscle cells—an indirect sign of intracellular oxidative damage—precedes lesion development in *Apoe* ^*-/-*^ mice [20]. Also, there is experimental evidence that links increased intracellular ROS levels, secondary to mitochondrial uncoupling in aortic smooth muscle cells, within the aorta to initiation of atherosclerosis [21]. Taken together, our results add support to the hypothesis that excessive intracellular ROS within the artery wall initiate atherosclerosis. In contrast, elevated extracellular ROS could only be detected in established atherosclerotic lesions and may not be involved in initiation of atherosclerosis. Thus, our results argues against the hypothesis that oxidative modification of LDL by extracellular ROS is an initiating event in atherosclerosis [22]. In agreement with our results, *Apoe* ^*-/-*^ mice that also lack the main extracellular ROS scavenging enzyme ec-SOD are not more prone to atherosclerosis than *Apoe* ^*-/-*^ mice with ec-SOD [23].

Both macrophages and smooth muscle cells, the most abundant cells in atherosclerotic lesions, produce ROS [1–3]. However, little is known about whether these cells produce intracellular or extracellular ROS. Our results indicate macrophages as the main source of intracellular ROS. High intracellular ROS production may help explain why lesion macrophages, that produce most ATP without oxygen [24], consume a lot of oxygen [25]. Lowering macrophage oxygen demand, by decreasing intracellular ROS, may improve lesion oxygenation and thus promote healing of lesions [24]. We identified smooth muscle cells as the main source of extracellular ROS within atherosclerotic lesions. This may have therapeutic implications because different treatment strategies are needed to target intracellular and extracellular ROS. For example, gene-therapy with extracellular antioxidant ec-SOD decreases restenosis in a rabbit model [26]. This agrees with our finding since oxidative stress in smooth muscle cells plays a key pathogenic role in restenosis.

Five days lipid-lowering by atorvastatin reduced ROS levels within established atherosclerotic lesions whereas more pronounced lipid-lowering by diet did not. The statin-induced reduction of ROS levels was not associated with decreased lesion inflammation. Thus, reduction in lesion ROS levels is an early, lipid-lowering independent, effect of atorvastatin. A lipid-lowering independent ROS reducing effect of statin treatment has previously been demonstrated in both smooth muscle cells [27] and macrophages *in vitro* [28]. However, the *in vivo* relevance of such *in vitro* studies is unclear. Cells in culture are grown on plastic and in excess of oxygen, nutrients and growth factors. In contrast, smooth muscle cells and macrophages in atherosclerotic lesions interact with an extracellular matrix and have limited access to oxygen, nutrients and growth factors [18, 24]. Thus, our observation that systemic statin treatment decrease ROS in intact lesions also under *in vivo* like conditions is important. The decrease in lesion ROS levels may be an additional beneficial effect of statin treatment, in addition to lipid lowering. Supporting our *ex vivo* observation in atherosclerotic vessels, previous studies in non-atherosclerotic aortas in hypertensive rats have shown a similar antioxidant effect of atorvastatin treatment [29]. Since excessive ROS promote inflammation and endothelial dysfunction [3], we speculate that the statin-induced decrease in ROS may help explain the anti-inflammatory and lesion stabilizing effects reported after long term (>4 weeks) statin treatment [13, 14, 19, 30].

We introduce a new technique to visualize intracellular and extracellular ROS within mouse aortas incubated under conditions resembling those *in vivo*. To this purpose, we used incubation conditions that maintain *in vivo-*like distribution of oxygen and nutrients within atherosclerotic lesions [18]. However, one potential limitation of our model is lack of intravascular flow. Intravascular flow around bifurcations and curvatures, predilection sites for atherosclerosis, are characterized by oscillating shear stress. Oscillating shear stress, in turn, is a potent stimulus for superoxide production and may be linked to atherosclerosis development [31]. This is one aspect of atherosclerosis development that may not be detected using our *ex vivo* model. Also, we used isoflurane to anesthetize mice and isoflurane increase ROS production *in vitro* [32]. Thus, it is possible that inhaled isoflurane may affect ROS levels in the aorta. However, since all animals were anesthetized in an identical manner, the differences reported here would still be valid.

## Conclusions

Using a new *ex vivo* imaging technique, we show that intracellular ROS increase early in atherosclerosis development, suggesting possible involvement in initiation of atherosclerosis. Analysis of advanced atherosclerotic lesions indicates that macrophages produce mainly intracellular ROS and smooth muscle cells mainly extracellular ROS. Furthermore, oral treatment with high dose atorvastatin decreased ROS levels within advanced atherosclerotic lesions in mice. The effect was independent of lipid lowering and not secondary to changing lesion inflammation or cell composition.
